# Development and Evaluation of an e-Learning Module for Low- and Middle-Income Countries on the Safe Handling of Chemotherapy Drugs

**DOI:** 10.1007/s13187-021-02113-z

**Published:** 2021-11-17

**Authors:** Sandrine von Grünigen, Berangère Dessane, Pauline Le Pape, Ludivine Falaschi, Antoine Geissbühler, Pascal Bonnabry

**Affiliations:** 1 Pharmacy, Geneva University Hospitals, Rue Gabrielle-Perret-Gentil 4, 1205 Geneva, Switzerland; 2 Institute of Global Health, Faculty of Medicine, University of Geneva, Geneva, Switzerland; 3 HI5lab, Department of Radiology and Medical Informatics, University of Geneva, Geneva, Switzerland; 4 Division of eHealth and Telemedicine, Geneva University Hospitals, Geneva, Switzerland; 5 Institute of Pharmaceutical Sciences of Western Switzerland, School of Pharmaceutical Sciences, University of Geneva, Geneva, Switzerland

**Keywords:** Safe handling practices, Cytotoxic drugs, Low- and middle-income countries, Chemotherapy, e-Learning

## Abstract

Despite the growing use of chemotherapy drugs in resource-constrained settings, training opportunities on safe handling practices are lacking. This study’s objectives were to develop and evaluate an e-learning training module on the safe handling of chemotherapy drugs to strengthen knowledge and practices in low- and middle-income countries (LMICs). The module’s curriculum was developed using the *Six-Step Approach for Curriculum Development for Medical Education*. Asynchronous, self-paced, e-learning lessons within the module were created and uploaded onto a free online platform, Pharm-Ed. The study ran online from January to April 2021. Participant recruitment was done using convenience sampling through various channels (social media, communities of practice). Training module effectiveness was evaluated using knowledge assessments (a pre-test and post-test study design) and participant satisfaction. We developed a comprehensive e-learning module on the safe handling of chemotherapy drugs comprising 11 asynchronous, self-paced, e-learning lessons. Eighty-two participants (68% pharmacists and 17% pharmacy students) from 17 countries completed at least one lesson, with a total of 259 lessons completed. Evaluation of the different lessons showed significant improvements in theoretical knowledge (*p* < 0.01) in all except one lesson and a high degree of participant satisfaction. As the use of anti-cancer drugs in LMICs will continue to increase, this e-learning module is an effective means to address the lack of training opportunities on the safe handling of chemotherapies for healthcare workers in these countries. The module could be integrated into a multi-modal approach aimed at reducing occupational exposure and increasing patient safety in cancer care centers.

## Introduction

In recent years, the use of chemotherapy drugs has increased tremendously in low- and middle-income countries (LMICs). Indeed, in response to the rising burden of cancer and its economic and human-related development threat, cancer management has become a priority for many LMICs [1–3]. Among various strategies and actions, the international community has made considerable efforts to improve patient access to anti-cancer medicines [4, 5]. Due to their inherent toxicity, however, these drugs require great precautions in handling and use [6]. Patient safety and occupational exposure have been areas of great concern for many years for the professional associations and national authorities in high-income countries [7–9]. Indeed, there are several reasons why cancer treatment management is a high-risk process: the complexity of treatment regimens, patient fragility, the very nature of the drugs, their administration routes, and so on. Over the years, numerous best practice guidelines and recommendations have been developed. Unfortunately, in countries where cancer management is more recent, the conditions for the safe use of chemotherapies are not always met [5, 10]. Several studies have reported safety risks, including insufficient knowledge, unsuitable infrastructure, the unavailability of materials, multitasking, work pressures, and high patient numbers [11, 12]. Other studies have reported that improper working practices were due to a lack of training, a lack of awareness, and false beliefs [13, 14]. As the GLOBOCAN statistics produced by the International Agency for Research on Cancer (IARC) predict a sharp increase in cancers by 2040, particularly in LMICs, more healthcare workers and more hospitals will be engaged in cancer care and chemotherapy drug use. It is thus imperative to take actions to promote and improve safe chemotherapy handling practices [1].

In recent years, taking advantage of information and communication technologies and developing e-learning strategies have been strongly encouraged for healthcare workers education [15, 16]. Distance education has grown significantly, particularly in LMICs, where there is a strong need to alleviate the shortage of trained, qualified professionals. One of e-learning’s many advantages is that it can transcend the geographical, political, and time barriers to education and thus extend training opportunities and access to larger numbers of people. Besides, technological progress in hardware and software and affordable internet connectivity have enabled broader technology access and usage in low-resource settings [17]. Distance education and e-learning are generic terms that include all kinds of educational methods, ranging from digital libraries to more complex distance learning networks and innovative methods such as virtual simulation or gamification [18]. However, achieving a real impact requires high-quality, relevant, and adaptable educational programs.

The present study’s objectives were to develop and evaluate an e-learning training module on the safe handling of chemotherapy drugs for strengthening knowledge and practices in LMICs.

## Methods

A steering committee created within the Geneva University Hospitals’ Pharmacy Department led the project and defined the module’s curriculum. It was composed of the department head, the pharmacist in charge of the cytotoxic drug preparation unit, and the study’s principal investigator. The module’s curriculum was developed based on the widely recognized and systematic *Six-Step Approach for Curriculum Development for Medical Education* [19].

### Curriculum and e-Learning Development

- *Step 1—Problem identification and needs assessment:* Needs assessment was based on an online survey evaluating the safe handling practices in many different settings in LMICs and on audits the authors conducted in four African hospitals (unpublished data) [10].
- *Step 2—Target audience:* The e-learning module was principally aimed at healthcare professionals (physicians, nurses, pharmacists, pharmacy technicians) handling chemotherapies and working in low-resource settings. The module was developed in French to target French-speaking LMICs.
- *Step 3—Goal and objectives:* The module’s goal was to cover the main aspects of the safe handling of chemotherapies all along the chemotherapy pathway (e.g., receiving drugs, storage, transport, prescription, preparation, administration, waste management, and disposal), to ensure patient safety, and reduce the risks of occupational exposure and environmental contamination. The learning objectives for each lesson within the module were set using Bloom’s Revised Taxonomy [20]. Lesson content was based on best practice guidelines and recommendations, and all content was reviewed and validated by the steering committee members before being published online. We followed the principle of constructive alignment to ensure coherence between the learning objectives, content, and the evaluation [21].
- *Step 4—Educational strategy:* The lessons were developed so that they could be followed using an asynchronous, self-paced learning format, meaning that participants can access, start, interrupt, and restart the different lessons at any time that suits their professional and personal schedule. Each lesson lasted from 10–30 min. e-Learning lessons were developed using the Articulate Storyline 3 (Articulate Global inc.) authoring tool, which enables publication in the HTML5 markup language. The module is thus compatible with most devices, including tablets and smartphones. To keep the lessons engaging and interactive, there are many embedded questions and answers with instantaneous feedback. Graphics such as stick figures were obtained from PresenterMedia® (Eclipse Digital Imaging Inc). Video tutorials were also filmed to better teach good practices in chemotherapy preparation and were then uploaded onto the Pharm-Ed YouTube channel.
- *Step 5—Implementation:* The entire e-learning module was subsequently uploaded onto the Pharm-Ed platform (www.Pharm-Ed.net), a collaborative online educational platform for promoting the efficient, safe, and rational management of medicines in hospitals. Access to the platform is free, but registration is required to participate in the e-learning module. The LearnDash® learning management system—a WordPress plugin—was used to manage and track the learning process.
- *Step 6—Evaluation and feedback:* We based our evaluation on the first two levels of Kirkpatrick’s training program evaluation model, i.e., reaction and learning [22]. At the end of each lesson, an online satisfaction questionnaire evaluated participants’ reactions, with satisfaction measured on 5-point Likert scales for various aspects of the lesson (content, courseware, level of difficulty, and overall satisfaction). The last part of the questionnaire contained open-ended questions on the lesson’s perceived strengths and weaknesses. Participants’ knowledge was assessed before (pre-test) and after (post-test) each lesson. These tests came in the form of multiple-choice questions that were identical for each lesson and integrated into our learning management system. The multiple-choice questions were developed to match the lesson’s pedagogical objectives and content (constructive alignment). The differences between the pre-test and post-test scores were used to calculate participants’ learning gain in each lesson.

### Conduct of the Study

This study used a one-group pre-test–post-test design. The e-learning module evaluation occurred from January to April 2021. Participant recruitment was done using convenience sampling via various channels, such as social media, communities of practice like the *e-med forum*, newsletters like *Pharm-Ed community*, and professional networking. Because the e-learning module was in French, only French-speaking participants were selected.

### Statistical Analysis

Data were exported from the LearnDash® learning management system to a Microsoft Excel® 2013 spreadsheet (Microsoft Corporation, Redmond, WA, USA). Participant characteristics were described using descriptive statistics. Participants’ pre-test and post-test score differences were assessed using the Wilcoxon signed-rank test. Statistics were calculated using R4.0.3 software (R Foundation for Statistical Computing, Vienna, Austria. https://www.R-project.org/).

## Results

The e-learning module encompassed 11 lessons covering the main aspects of the safe handling of chemotherapy drugs: (1) risks related to chemotherapy drugs, (2) logistical aspects specific to chemotherapy drugs, (3) safe chemotherapy prescription practices, (4) premises, (5) biosafety cabinets and isolators, (6) personal protective equipment, (7) ensuring preparation process safety, (8) ensuring chemotherapy administration safety, (9) incident management, (10) extravasations, (11) waste management. The average duration of the lessons varies between 10 to 30 min and the number of multiple choice questions in the pre/post-tests between five and twelve.

### Participants

Of the 125 participants, 82 (66%) completed the pre-test and post-test for at least one lesson. The other 43 participants did not complete a lesson, as they filled in either only the pre-test or the post-test. In total, 259 lessons were completed (an average of 3 lessons per participant), and 82 incomplete lessons were excluded (i.e., only the pre-test or post-test was filled in). Participants came from 17 countries and most were pharmacists (68%) or pharmacy students (17%) (Table 1).

**Table 1:** Participants’ characteristics

	No. of participants	No. of lessons
Countries		
Algeria	11 (13%)	33 (12.7%)
Belgium	1 (1%)	1 (0.4%)
Benin	1 (1%)	2 (0.8%)
Burkina Faso	1 (1%)	2 (0.8%)
Cameroon	3 (4%)	15 (5.8%)
Canada	1 (1%)	1 (0.4%)
Cote d’Ivoire	1 (1%)	1 (0.4%)
Democratic Republic of Congo	3 (4%)	4 (1.5%)
France	17 (21%)	45 (17.4%)
Gabon	1 (1%)	1 (0.4%)
Greece	1 (1%)	2 (0.8%)
Guinea	1 (1%)	2 (0.8%)
Madagascar	1 (1%)	2 (0.8%)
Mauritania	1 (1%)	2 (0.8%)
Morocco	10 (12%)	40 (15.4%)
Senegal	20 (24%)	90 (34.8%)
Tunisia	8 (10%)	16 (6.2%)
Profession		
Pharmacist	56 (68%)	179 (69.1%)
Pharmacy student	14 (17%)	38 (14.7%)
Pharmacy technician	3 (4%)	3 (1.2%)
Nurse	5 (6%)	33 (12.7%)
Physician	1 (1%)	1 (0.4%)
Other	1 (1%)	1 (0.4%)
Unknown	2 (2%)	4 (1.5%)
Type of institution		
University teaching hospital	19 (23%)	62 (23.9%)
Regional hospital	4 (5%)	15 (5.8%)
District hospital	4 (5%)	19 (7.3%)
Military hospital	3 (4%)	4 (1.5%)
Private institution	4 (5%)	18 (6.9%)
Student	14 (17%)	37 (14.3%)
Other (NGO, health ministry, university)	13 (16%)	39 (15.1%)
Unknown	21 (26%)	65 (25.1%)
Total	**82**	**259**

### Effectiveness on Improvements in Knowledge

**Fig. 1:**
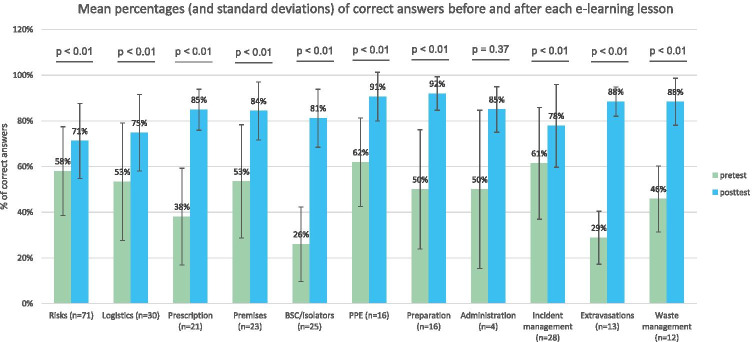
Pre-test and post-test results for each lesson expressed as mean percentage of correct answer with standard deviation

Figure 1 shows the mean pre-test and post-test scores for each lesson. In general, participants’ pre-test knowledge levels were mostly moderate across the different lessons (i.e., around 50% of answers correct), except for three lessons where baseline knowledge was lower: prescribing (37%), biosafety cabinets and isolators (26%), and extravasations (29%). Post-test results showed significant improvements in knowledge (*p* < 0.01) after all the lessons except for the one on ensuring chemotherapy administration safety, where the number of participants was too low to detect any potential effect (*n* = 4).

### Satisfaction

**Fig. 2:**
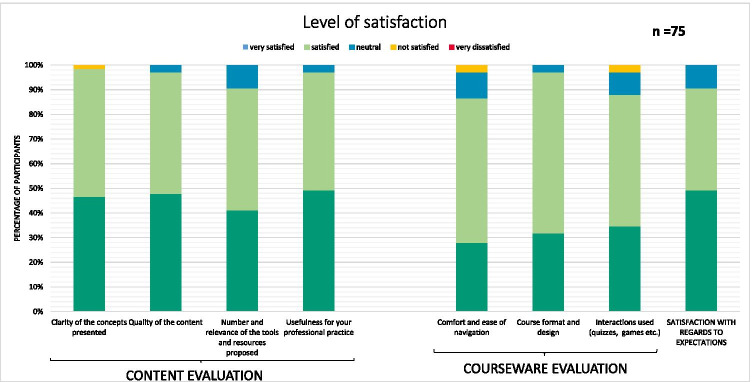
Participants’ reported levels of overall satisfaction (for all lessons)

Of the 259 completed lessons, only 75 (29%) were accompanied by their respective completed satisfaction forms, of which 38 (51%) had been filled in by pharmacists, 25 (33%) by pharmacy students, 6 (8%) by pharmacy technicians, and 5 (7%) by nurses. All the lessons received feedback, but the majority of the results concerned the lessons on the risks related to chemotherapy drugs (43%) and the logistical aspects specific to chemotherapy drugs (21%). Overall, participants expressed a high level of satisfaction with the course content and the courseware (Fig. 2). Almost every participant (99%) would have recommended the lesson to a colleague. The level of difficulty of the concepts presented and the tests were considered “appropriate” on the majority of the forms (81% and 85%, respectively), whereas very few reported that the level of difficulty was too low (15% and 7%, respectively) or too high (1% and 4%, respectively).

## Discussion

### Summary of Results

We developed a comprehensive e-learning module on the safe handling of chemotherapy drugs comprising 11 asynchronous, self-directed e-learning lessons. The evaluation of these different lessons revealed significant improvements in participants’ theoretical knowledge in all but one lesson (which had insufficient statistical power) and a high degree of participant satisfaction in terms of content and courseware. In general, the post-test scores for the different lessons were relatively high. These findings reflect the positive effects of the constructive alignment principle that we followed in the module’s development phase. Lessons with slightly lower post-test scores (e.g., for risks and logistics) will therefore be reviewed in more detail to verify their constructive alignment. Future multiple-choice questions for the tests will be pilot-tested to detect any issues related to their structure or formulation.

### Comparison with the Relevant Literature

As with the majority of the studies included in the systematic review by Barteit et al. (2019), we used a pre-test and post-test design to assess participants’ knowledge using multiple-choice questions [17]. Although several studies have assessed nurses’ knowledge and practices in the safe handling of cytotoxic drugs in LMICs, our study is, to the best of our knowledge, the first to evaluate an e-learning module on the safe handling of chemotherapy drugs in these countries [11, 12, 23, 24]. Indeed, our study participants were also predominantly pharmacists (89%), with only 6% being nurses. This difference was due to the communication channels used to build our convenience sample: these did not permit large numbers of nurses to be reached. The participation of a larger number of nurses would have been desirable, however, as they are directly concerned by many of the aspects addressed in this module.

### Strengths and Limitations

This study involves participants from a wide diversity of settings, not only geographically but also in terms of working environments. The high level of satisfaction thus reflects the module’s adaptability and applicability across these different contexts. Making these courses available in additional languages, such as English and Spanish, could be very beneficial to healthcare workers in other LMICs where these languages are spoken.

The present study had some limitations, including the lack of a control group. In addition, the sample size differed from lesson to lesson and, for some, the number of participants was relatively low, making it difficult to analyze sub-groups or interpret results. Because not all of the participants completed the entire e-learning module, it was impossible to measure its overall effectiveness. Regarding the individual lessons, we limited our evaluation to the Kirkpatrick model’s first two levels, namely user satisfaction and knowledge acquired; the competencies evaluated in levels 3 and 4—resulting from the transposition of knowledge into professional practice and its impact at the institutional level—were not assessed. In addition, we assessed knowledge directly after each lesson in the training module. It would be interesting to study knowledge retention over time.

### Implications for Practice

The present study demonstrated some of the effectiveness and appropriateness of this e-learning module for healthcare professionals in LMICs. Implementing this type of training for all the staff involved in handling chemotherapies should become mandatory as it could help reduce the risks of occupational exposure and improve patient safety. To the best of our knowledge, there are very few opportunities for training healthcare workers in this field in resource-limited countries. This training module can be followed for free on the Pharm-Ed e-learning platform. It is a beneficial and appropriate training module for a variety of settings. It could also be integrated into a blended learning approach with one or more face-to-face modules used to emphasize specific skills and behaviors; it could encourage the exchange of best practices during focus groups and the discussion of barriers to institutional change. However, although improving individual knowledge levels and behaviors is essential, the adoption of safety measures and the application of safe practices at the institutional level require broad changes in the approach to workplace safety. Using a multi-modal approach should include implementing recommended safety policies and procedures, the availability of safety equipment, knowledge reinforcement, supervision, and managerial support for safety programs [25].

### Future Research

To address some of the present study’s limitations, it would be interesting to retest the participants’ knowledge several months after their training to measure knowledge retention over time. Secondly, to complete the evaluation of the module’s effectiveness, a field study involving the participating healthcare institutions could be conducted to observe actual changes in behaviors and practices: this would represent the third level in Kirkpatrick’s evaluation model for training programs. The use of other qualitative methods, such as in-depth interviews and focus groups, could help to investigate the barriers and facilitators to improving practices.

## Conclusion

The systematic and thorough approach used in the development of this training module led to very positive results, not only in terms of participant satisfaction but also in terms of their improved knowledge. The results from the satisfaction questionnaire underlined the e-learning module’s relevance and appropriateness in terms of content and format. The significant improvements in knowledge measured for most of the e-learning lessons partly reflect their effectiveness. As the use of anti-cancer drugs will only continue to increase in LMICs, this e-learning module provides a free, simple, easily accessed means of addressing the lack of training opportunities on the safe handling of chemotherapies for healthcare workers in these countries. It could be integrated into a multi-modal approach to reducing occupational exposure and increasing patient safety in cancer care centers.

## Data Availability

Training material is available on www.Pharm-Ed.net.
